# Septic Peritonitis from Ruptured Pyometra: Case Report and Literature Review

**DOI:** 10.1002/ccr3.72517

**Published:** 2026-04-11

**Authors:** Chernet T. Mengistie, Biruk T. Mengistie, Selam D. Temesgen, Kalab Y. Gete, Biruk Z. Bekele, Elias G. Bayu, Sileante M. Tamru

**Affiliations:** ^1^ School of Medicine, College of Health Sciences Addis Ababa University Addis Ababa Ethiopia; ^2^ Department of Obstetrics and Gynecology, College of Health Sciences Addis Ababa University Addis Ababa Ethiopia; ^3^ School of Medicine College of Medicine and Health Sciences, Bahir Dar University Bahir Dar Ethiopia; ^4^ EPIC Health Systems Addis Ababa Ethiopia

**Keywords:** peritonitis, postmenopausal, pyometra, sepsis, uterine perforation

## Abstract

Pyometra, pus within the uterine cavity due to cervical obstruction, is uncommon in postmenopausal women, and spontaneous uterine rupture from pyometra is exceedingly rare but may cause severe peritonitis and sepsis; we report a 57‐year‐old postmenopausal woman who presented with foul vaginal discharge, progressive lower abdominal pain, fever, hypotension, and ultrasound evidence of turbid intraperitoneal fluid, whose contrast CT demonstrated an endometrial fluid collection with a defect in the posterior uterine wall communicating with the peritoneal cavity; despite initial resuscitation and broad‐spectrum antibiotics she deteriorated and underwent urgent exploratory laparotomy, which revealed ~1500 mL of purulent ascites and a 3.0‐cm posterior uterine wall perforation, she had a total abdominal hysterectomy with bilateral salpingo‐oophorectomy and thorough peritoneal lavage, histopathology confirmed necrotizing endometritis/pyometra without malignancy, and her postoperative recovery was uncomplicated with discharge on postoperative day 9; this case highlights the diagnostic challenge of ruptured pyometra, the value of CT when rupture is suspected, and the necessity of early surgical source control combined with antibiotics to improve outcomes.

## Key Clinical Message

Spontaneous uterine perforation from pyometra is a rare but life‐threatening cause of acute abdomen in postmenopausal women. Early imaging, aggressive resuscitation, broad‐spectrum antibiotics, and prompt surgical source control are essential. The prognosis is significantly better in the absence of underlying malignancy.

## 1 Introduction

Pyometra is defined as the collection of purulent fluid within the uterine cavity, usually resulting from obstruction of the cervical canal [1]. It is a rare condition in women overall; reported incidence estimates range from 0.01% to 0.5% among gynecologic admissions [2]. The condition most often affects elderly women; one series found a median age of presentation of 65 years [3]. Cervical stenosis from senile atrophy, inflammation, or previous surgery predisposes to fluid buildup [2]. Importantly, underlying genital tract malignancies are a well‐recognized cause of pyometra. Approximately 20%–25% of cases are associated with malignancy [1, 4]. Other causes include benign lesions such as endometrial polyps, leiomyomas, or chronic endometritis [4]. In many cases, no specific etiology is identified [1].

Clinically, patients with pyometra may present with a classic triad of lower abdominal or pelvic pain, purulent vaginal discharge, and postmenopausal bleeding [1]. However, this triad is not always fully present, and early symptoms can be nonspecific (e.g., vaginal irritation or discharge). Some patients may even be asymptomatic until complications occur [5]. The risk of severe complications is high. As the intrauterine pressure rises, spontaneous perforation of the uterine wall can occur, leading to generalized peritonitis [6]. Pyometra‐related sepsis, bacteremia, and septic shock have all been reported [7, 8, 9]. Notably, cases without associated malignancy tend to have a better prognosis than those with cancer [1].

Diagnosis of pyometra and its complications relies on imaging [2]. Transvaginal or abdominal ultrasound is typically the first‐line evaluation and can detect an enlarged uterus filled with echogenic fluid [2, 10]. However, ultrasound has limited sensitivity for detecting uterine rupture [10]. In suspected perforation, cross‐sectional imaging is important. CT or MRI can reveal the extent of fluid collections and may show a defect in the uterine wall or gas in the peritoneal cavity [2]. Despite these tools, the preoperative diagnosis of pyometra perforation is challenging; only a minority of cases are recognized before surgery [11, 12]. Surgical management is required in complicated cases [7]. For nonruptured pyometra, cervical dilatation, and drainage with broad‐spectrum antibiotics is the standard approach [1]. If malignancy is suspected or identified, definitive surgery (hysterectomy) is usually indicated [1, 2]. In cases of perforation with peritonitis, total abdominal hysterectomy with bilateral salpingo‐oophorectomy and copious peritoneal lavage is generally advocated [11, 13]. Herein, we report a case of spontaneous uterine perforation secondary to pyometra, highlighting diagnostic pitfalls and the necessity of prompt surgical source control.

## 2 Clinical History/ Examination

A 57‐year‐old para‐IV postmenopausal woman, amenorrheic for 20 years, presented with a one‐week history of foul‐smelling vaginal discharge, progressive lower abdominal pain, anorexia, nausea, and low‐grade intermittent fever. On initial evaluation, she appeared acutely unwell and in pain. Vital signs were: temperature 37.8°C, pulse 120 beats/min (feeble), blood pressure 85/54 mmHg. Abdominal examination revealed suprapubic and lower abdominal tenderness on deep palpation without a palpable mass or organomegaly. Pelvic examination showed a closed cervical os and bilateral adnexal and cervical motion tenderness without visible vaginal bleeding or discharge.

## 3 Differential Diagnosis, Investigations, and Treatment

Initial investigations demonstrated leukocytosis (WBC 17.4 × 10^3^/μL, neutrophils 89.6%). Abdomino‐pelvic ultrasound reported turbid intraperitoneal and pelvic fluid, suggesting an abscess, with otherwise normally appearing abdominal and pelvic organs.

The patient was admitted with a working diagnosis of complicated pelvic inflammatory disease and sepsis of presumed genitourinary origin. The patient was started on empirical intravenous ceftriaxone (1 g every 12 h) plus metronidazole (500 mg every 8 h) along with fluid resuscitation. Despite therapy, the clinical picture failed to improve over the next 48–72 h; she developed worsening nausea and persistent vomiting (5–6 episodes/day), and inflammatory markers rose. Repeat blood tests showed progressive leukocytosis (WBC 21.5 × 10^3^/μL).

**FIGURE 1:**
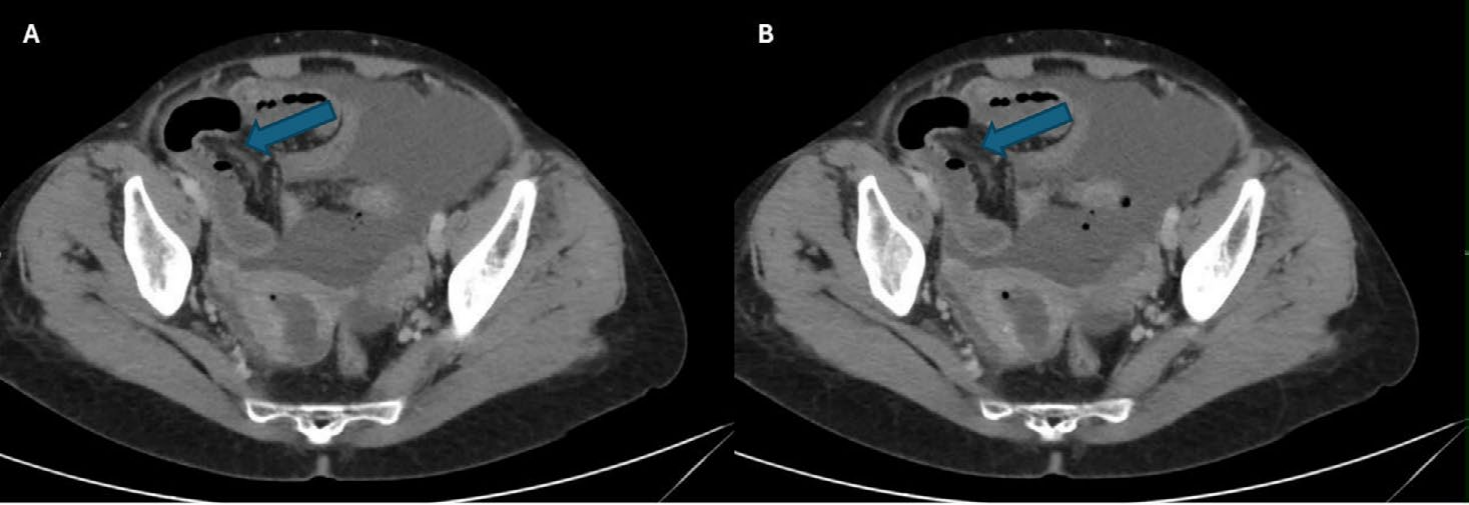
(A–B) Axial contrast‐enhanced CT images of the pelvis (10 March 2024) showing loculated intraperitoneal fluid with internal heterogeneous debris and small gas foci, with loss of posterior uterine fundal contour and a focal defect communicating with the collection.

Repeat ultrasound showed moderate ascites with thick internal echogenic debris, thickened peritoneum and a heterogeneous uterus with a dilated adnexal tubular lesion. Contrast CT of the abdomen and pelvis demonstrated moderate nondependent peritoneal fluid with multiple internal foci, endometrial fluid collection, and a defect in the posterior uterine wall communicating with the peritoneal cavity; enlarged adnexa and reactive pelvic and para‐aortic lymph nodes were also noted (Figure 1).

**FIGURE 2:**
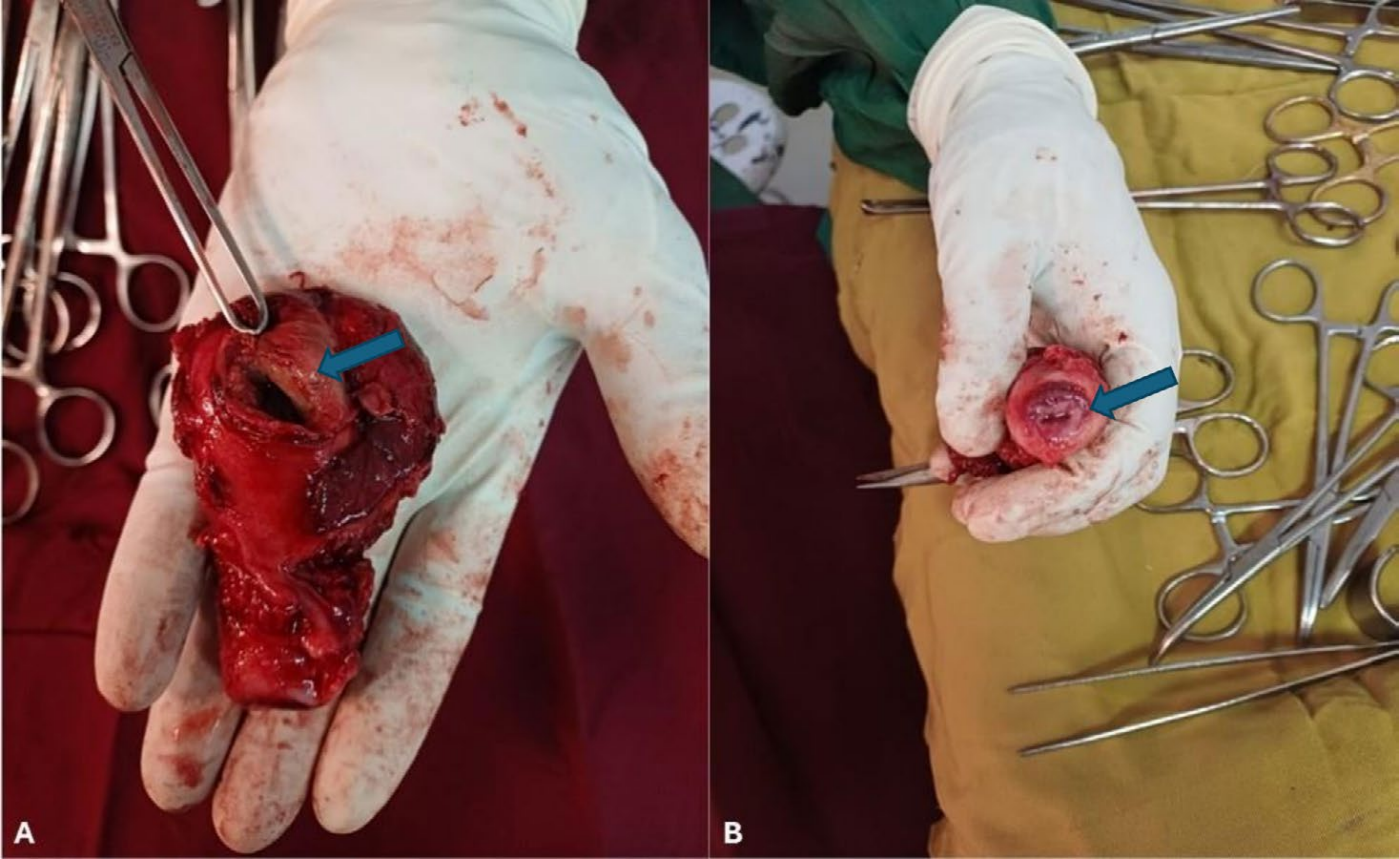
Resected uterus (A, external view; B, cross‐section) demonstrating a 3.0‐cm posterior uterine wall perforation with necrotic myometrium and adherent purulent material consistent with perforated pyometra.

Given the imaging findings and the patient's deteriorating condition, a diagnosis of uterine perforation with peritonitis and abdominopelvic collection was made, and the patient underwent exploratory laparotomy. Operative findings included approximately 1500 mL of thick purulent intraperitoneal fluid, a 3‐cm perforation in the posterior uterine wall, dense peritoneal and bowel adhesions, and tubo‐ovarian adhesions (Figure 2). A total abdominal hysterectomy with bilateral salpingo‐oophorectomy and thorough peritoneal lavage was performed. Specimens and peritoneal pus were submitted for histopathology and microbiology. Microbiologic analysis (Gram stain, culture, and sensitivity) was performed; however, the results were not contributory.

Peritoneal fluid cytology showed a suppurative effusion with abundant neutrophils. Histopathology confirmed pyometra with acute on chronic necrotizing inflammation extending into the myometrium and focal necrosis consistent with uterine perforation.

## 4 Outcome and Follow‐Up

Postoperatively, her immediate recovery was uncomplicated: drain output was minimal, vital signs normalized, and inflammatory markers declined gradually (WBC 10.8 × 10^3^/μL). She was discharged in stable condition on postoperative day 9 with outpatient follow‐up.

## 5 Discussion

Spontaneous uterine perforation secondary to pyometra is an exceedingly rare cause of acute abdomen [2, 4]. Worldwide, only a few dozen cases of ruptured pyometra have been reported in modern literature. For example, a 2016 review by Yin et al. identified only ~80 cases between 1949 and 2015 and reported a mortality rate of 31.9% [11]. In sub‐Saharan Africa, documented cases are especially scarce. Recently, Kishe et al. (Tanzania, 2025) described a postmenopausal woman with pyometra‐related uterine perforation, highlighting the need for vigilance in this setting [14].

The clinical presentation of perforated pyometra often mimics other causes of peritonitis [2]. Patients typically present with acute, diffuse abdominal pain, fever, and signs of sepsis [7]. Because symptoms are nonspecific, preoperative diagnosis is challenging [2]. Computed tomography in our patient showed free fluid, uterine wall defect, and fluid in the endometrial cavity communicating with the peritoneum, which are key findings suggestive of uterine rupture by pyometra. However, in many reported cases, the diagnosis was only made intraoperatively [1, 2, 11, 13]. In one series, only about 21% of cases had the correct diagnosis before surgery [11]. Notably, physical exam findings can include a closed, stenotic cervix and palpable uterine enlargement, which may prompt consideration of pyometra [2]. The presence of foul vaginal discharge in an elderly woman is another important clue [2, 14]. Imaging features that distinguish a perforated pyometra from gastrointestinal perforation include a dilated, fluid‐filled uterus with wall disruption and absence of bowel pathology [2, 10]. When these findings are present in the appropriate clinical context, pyometra rupture should be suspected.

Treatment of perforated pyometra must be prompt and definitive. Broad‐spectrum antibiotics and resuscitation are essential first steps [7, 11]. Surgical management usually involves hysterectomy and salpingo‐oophorectomy with peritoneal lavage [11]. In most reported cases, a total hysterectomy was performed at laparotomy [2, 7, 10]. This was also the management in our patient, and she recovered without complications. A hysterectomy eliminates the source of infection and allows thorough decontamination of the peritoneal cavity [7, 10]. In rare circumstances, more conservative approaches (such as drainage and delayed surgery) have been attempted when patients are critically unstable [13], but these carry significant risks. In general, early definitive surgery is favored for better outcomes [2, 7, 11].

Outcomes in perforated pyometra are guarded. The literature shows high morbidity and mortality, reflecting the severity of diffuse peritonitis in often frail patients [7, 11]. Poor prognostic factors include advanced age, delayed diagnosis, and concomitant malignancy [1, 11]. By contrast, patients without malignancy, like ours, tend to fare better [1]. Following surgery and antibiotics, many patients recover, but the illness is life‐threatening [2, 7]. Long‐term follow‐up is recommended, as pyometra can recur in patients treated conservatively [1].

## 6 Conclusion

Spontaneous perforation of pyometra is a rare but serious cause of acute abdomen in postmenopausal women. This case highlights key learning points: clinicians should consider pyometra rupture in elderly females with acute abdominal pain, sepsis, and a history of vaginal discharge. A high index of suspicion and prompt imaging (ultrasound and CT) are crucial for diagnosis. Definitive management generally requires surgical intervention (often hysterectomy with salpingo‐oophorectomy and peritoneal lavage) combined with broad antibiotics. Early recognition and aggressive treatment can significantly improve outcomes, especially when no malignancy is present. Awareness of this rare entity can prevent diagnostic delays and reduce the risk of catastrophic complications in similar patients.

## Author Contributions

**Chernet T. Mengistie:** conceptualization, writing – original draft, writing – review and editing. **Biruk T. Mengistie:** visualization, writing – original draft, writing – review and editing. **Selam D. Temesgen:** resources, writing – original draft. **Kalab Y. Gete:** data curation, writing – review and editing. **Biruk Z. Bekele:** data curation, resources. **Elias G. Bayu:** resources. **Sileante M. Tamru:** supervision, visualization.

## Funding

The authors have nothing to report.

## Ethics Statement

IRB review and approval were waived for this case report.

## Consent

Written informed consent for publication of the clinical details and accompanying images was obtained; the signed consent form is held by the corresponding author and can be made available to the Editor on request.

## Conflicts of Interest

The authors declare no conflicts of interest.

## Data Availability

The data underlying the results presented in this work are available within the manuscript.
